# New Insights into Mitochondria in Health and Diseases

**DOI:** 10.3390/ijms25189975

**Published:** 2024-09-16

**Authors:** Ya Li, Huhu Zhang, Chunjuan Yu, Xiaolei Dong, Fanghao Yang, Mengjun Wang, Ziyuan Wen, Mohan Su, Bing Li, Lina Yang

**Affiliations:** Department of Genetics and Cell Biology, Basic Medical College, Qingdao University, Qingdao 266071, China; liyaemail2022@163.com (Y.L.); ts_zhh2580@163.com (H.Z.); 17861281992@163.com (C.Y.); dongxiaoleiqd@163.com (X.D.); yfhao1996@163.com (F.Y.); wangmj90405@163.com (M.W.); 13287377676@163.com (Z.W.); smh13731390822@163.com (M.S.); libing_516@qdu.edu.cn (B.L.)

**Keywords:** mitochondrial, bioenergetics, ROS, aging, mtDNA, mutations, mitochondrial dysfunction, mitochondrial targeted therapy

## Abstract

Mitochondria are a unique type of semi-autonomous organelle within the cell that carry out essential functions crucial for the cell’s survival and well-being. They are the location where eukaryotic cells carry out energy metabolism. Aside from producing the majority of ATP through oxidative phosphorylation, which provides essential energy for cellular functions, mitochondria also participate in other metabolic processes within the cell, such as the electron transport chain, citric acid cycle, and β-oxidation of fatty acids. Furthermore, mitochondria regulate the production and elimination of ROS, the synthesis of nucleotides and amino acids, the balance of calcium ions, and the process of cell death. Therefore, it is widely accepted that mitochondrial dysfunction is a factor that causes or contributes to the development and advancement of various diseases. These include common systemic diseases, such as aging, diabetes, Parkinson’s disease, and cancer, as well as rare metabolic disorders, like Kearns–Sayre syndrome, Leigh disease, and mitochondrial myopathy. This overview outlines the various mechanisms by which mitochondria are involved in numerous illnesses and cellular physiological activities. Additionally, it provides new discoveries regarding the involvement of mitochondria in both disorders and the maintenance of good health.

## 1. Introduction

**Figure 1:**
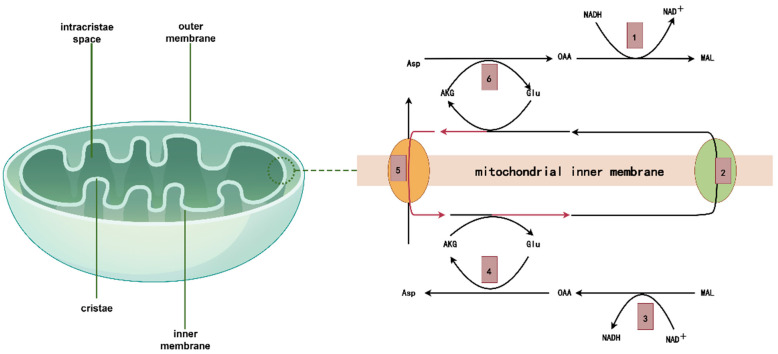
Mitochondrial structure and the process by which NADH + H^+^ enters the mitochondria via specific shuttling in the inner mitochondrial membrane. The process of producing ATP is intricate. For instance, the metabolism of glucose involves the cytoplasmic glycolysis process, the mitochondrial matrix TCA cycle, and oxidative phosphorylation accompanied by the production of ATP. Of these, the pyruvate transporter facilitates the entry of pyruvate generated during glycolysis into the mitochondria, but the mechanism of NADH + H+ entering the mitochondria is more intricate: 1. NADH + H^+^ in the cytoplasm is treated by malate dehydrogenase to make oxaloacetic acid (OAA) accept 2 H and become malic acid (MAL). 2. Malic acid enters mitochondria via transport carriers in the inner membrane.3. Under the action of malic acid entering mitochondria, NAD^+^ is used as acceptor to form oxaloacetic acid and NADH + H^+^. 4. Oxaloacetic acid and glutamic acid are transformed into aspartic acid and alpha-ketoglutaric acid by the interaction of glutamic acid with glutamic acid through glutamic oxaloacetic acid transaminase. 5. Aspartate (Asp) and α-ketoglutaric acid enter the cytoplasm with the help of mitochondrial transport carriers. 6. Glutamate (Glu) consumed in the mitochondria is supplemented by the exchange of glutamate in cellular fluid and outgoing aspartic acid through the reverse glutamate–aspartic acid transport carrier.

Mitochondria are semi-autonomous organelles present in all eukaryotic cells except for adult mammalian red blood cells. They are commonly known as the “powerhouses” of the cell due to their ability to perform oxidative phosphorylation, a process that generates energy. Typically, mitochondria have an elliptical or rod-shaped structure and are enveloped by two membranes: an inner membrane and an outer membrane. The cristae, which have intricate folding patterns on the inner membrane, enhance the available surface area for efficient oxidative phosphorylation (Figure 1). mtDNA is a circular molecule with two strands that contains the genetic information for many subunits of the respiratory chain complex and mtDNA molecules. The processes of mtDNA transcription, translation, and replication operate autonomously, without being influenced by the actions of nuclear DNA. Mitochondria are involved in Ca^2+^ regulation, apoptosis, and other metabolic activities, such as the citric acid cycle and fatty acid oxidation. Many diseases have been associated with mitochondria, including diabetes, Parkinson’s disease, and Alzheimer’s disease. To improve human health and enable mitochondrial targeted therapy for diseases, it is helpful to comprehend the intricate role that mitochondria play in cell activity and to identify the mechanisms underlying mitochondrial malfunction linked to various diseases.

## 2. Oxidative Phosphorylation and Energy Production

The main constituent of cellular metabolism is the mitochondrial oxidative phosphorylation system. The respiratory chain of the mitochondria consists of two mobile electron carriers and five enzyme complexes. The electron transport chain (ETC) drives ATP generation by integrating the reduction–oxidation process within the mitochondria, generating a proton gradient, and then dissipating it across the inner mitochondrial membrane. The electron transport chain in mitochondria is composed of Complex I, II, III, IV, and V, as well as the electron carrier coenzyme Q (CoQ) and cytochrome c [1]. Complex I is the initial site where electrons derived from NADH are introduced into ETC. It enhances the protonmotive force by transporting four protons across the inner mitochondrial membrane for every NADH oxidized. In addition, Complex I simultaneously manufactures reactive oxygen species (ROS) in a complex manner [2]. Complex II does not actively transport protons across the membrane like Complex I does. However, it does transfer electrons from FADH2 to CoQ. Complex III facilitates the transfer of electrons from CoQ to cytochrome c by actively moving protons across the membrane. Cytochrome C transfers electrons to Complex IV, facilitating the conversion of molecular oxygen into water. It achieves this by actively transporting protons across the membrane. Complex V, also known as ATP synthase, uses the protons produced by complexes I, III, and IV to convert ADP into ATP [3]. Abnormal oxidative phosphorylation and mitochondrial energy generation are associated with various diseases, including neurodegenerative disorders, myopathy, visual atrophy, and deafness. For example, mutations in the genes responsible for encoding the ETC component can lead to illnesses, such as dementia. This occurs by reducing the quantities of biological energy in the cell and disrupting the balance of the NAD^+^/NADH ratio in the mitochondria [4]. In addition, ROS, which plays a crucial role in cellular signal transmission, cell cycle regulation, and cell growth, is produced as a by-product of the ETC. The excessive creation of ROS also leads to cellular damage, which plays a significant role in the progression of several cancers and neurological illnesses [5].

### 2.1. mtDNA Mutations and Diseases

Anderson completed the process of determining the order of all the genetic material in the human mtDNA genome in 1981. mtDNA is a double-stranded circular DNA molecule that is 16.5 kb in length and has a relatively small molecular weight [6,7]. Mitochondria are extensively spread within cells. Each mitochondrion has a range of from 2 to 10 copies of mtDNA. mtDNA consists of both coding and non-coding sections. The coding region displays a high degree of sequence conservation and homology across multiple species. The number of genes in this area is 37. There are a total of 22 genes that encode tRNA in mitochondria. These tRNAs are responsible for recognizing and binding to all the codons involved in the translation of mitochondrial proteins. Additionally, there are two genes that encode the rRNA components of mitochondrial ribosomes, specifically the 16S and 12S rRNAs. The genes COX Ⅰ, COX Ⅱ, and COX Ⅲ are responsible for encoding subunits of the mitochondrial oxidative phosphorylation (OXPHOS) enzyme complex. These subunits form the catalytic active core of the cytochrome c oxidase (COX) complex, also known as Complex Ⅳ. The three components bear a resemblance to the cytochrome c oxidase seen in bacteria. The sequence of Complex Ⅴ’s F0 component, which consists of two subunits, has remained mostly unchanged throughout the process of evolution. The NADH-CoQ reductase complex (Complex I) consists of subunits ND1, ND2, ND3, ND4L, ND4, ND5, and ND6 [7,8,9]. On the other hand, a subunit in the CoQH2-cytochrome c reductase complex (Complex Ⅲ) encodes cytochrome b. Nevertheless, the production of nDNA encoded proteins in the cytoplasm and its subsequent entry into the mitochondria through a specific transport mechanism is essential for most of the protein components of mitochondria, as well as the proteins responsible for maintaining the structure and function of mitochondria. In addition, nDNA restricts the activity of mtDNA genes, and both nDNA and mtDNA need to work together to construct and sustain the mitochondrial oxidative phosphatase system [10]. Consequently, irregular production of proteins in the mitochondria and incorrect utilisation of cellular energy occur due to anomalies in both the mtDNA and nDNA genes. Mitochondrial diseases caused by mtDNA or nDNA abnormalities are hereditary [11]. These abnormalities affect the oxidative phosphorylation process of the mitochondrial respiratory chain, leading to issues with energy metabolism. Due to the varied activities of nDNA and mtDNA within cells, illnesses resulting from mutations in either kind of DNA exhibit distinct characteristics (Table 1).

### 2.2. mtDNA Diseases

Heterogeneity refers to the presence of both wild-type and mutant mtDNA in different proportions, which is a defining feature of mitochondrial diseases [12]. Many mtDNA mutations can lead to the occurrence of a particular disease, whereas a single mutation can give rise to different clinical presentations. The occurrence of these variants is often associated with the location of tissues and the abundance of the mutant mtDNA in the body. Consequently, the lesions are predominantly seen in the central nervous system (mitochondrial encephalopathy), skeletal muscle (mitochondrial myopathy), and both the central nervous system and skeletal muscle (mitochondrial encephalomyopathy). Leber’s hereditary optic neuropathy (LHON) and myoclonic epilepsy with red fiber ragged (MERRF) are the prevailing illnesses resulting from mutations in mtDNA). The clinical presentation of LHON is characterized by acute or subacute bilateral central vision loss resulting from substantial bilateral optic nerve atrophy. Concomitant system illnesses including the neurological system, heart, skeletal muscle, and other tissues may also be present. The three most frequent variations associated with LHON are G11778A, T14484, and G3460A. These mutations affect the ND1, ND4, and ND6 subunits of NADH dehydrogenase, respectively. The variants G11778A, G3460A, and T14484C were the most widespread, accounting for 56%, 31%, and 6.3% of all mutations, respectively [13,14]. The main clinical symptoms of MEBRF are paroxysmal epilepsy and increasing nervous system abnormalities, including mental impairment, ataxia, and intentional tremor. The patient has coarse, chaotic muscular fibers, along with an abnormal mitochondrial structure that accumulates within skeletal muscle cells. The term “broken red fibre” refers to the red coloration that occurs as a result of Gomori trichrome staining. MERRF can be caused by many mutations in the mtDNA gene, with the most common one being the mtDNA encoding tRNA gene 8344 region A>G (MTTK*MERRF8344G). This mutation leads to a deficit of enzyme complexes in the respiratory chain, specifically OXPHOS complex I and complex IV. The mutation causes the tRNA to separate from the ribosome. The synthesis of the chemical resulted in the deterioration of OXPHOS function, leading to multi-system impairments in the patients [15,16].

### 2.3. nDNA Diseases

Mitochondrial disorders can also arise from nuclear DNA (nDNA), which encodes the majority of the proteins necessary for proper mitochondrial function. These mutations can affect several cellular activities, including the production, import, and assembly of mitochondrial proteins. In addition, they adhere to prevalent inheritance patterns, such as autosomal dominant or recessive. Leigh syndrome is one of the disorders caused by mutations in nDNA. Leigh syndrome often presents clinically during the period between three months and two years of age. It is characterized by symptoms, such as motor skill regression, feeding difficulties, vomiting, irritability, and seizures. As the condition deteriorates, hypotonia, episodes of lactic acidosis, and generalized weakness may arise, potentially impacting renal and respiratory function. Neuroimaging, particularly MRI, can reveal symmetrical lesions in the brain stem or basal ganglia. Leigh syndrome patients generally have a grim prognosis, with the majority of youngsters succumbing to the ailment by the age of three [17]. However, there have been a few rare cases where the onset of the disease occurred later, and where its course was slower. The molecular aetiology of Leigh syndrome is attributed to mutations in nuclear genes that encode components of the oxidative phosphorylation pathway. These anomalies may affect the pyruvate dehydrogenase complex, coenzyme Q10 metabolism, and the mitochondrial respiratory chain. Leigh syndrome often arises from mutations in the SURF1 gene, which plays a role in the formation of cytochrome c oxidase, an essential enzyme in the mitochondrial respiratory chain. Despite the discovery of multiple causal mutations, Leigh syndrome remains incurable, and existing treatments often yield limited results. The primary focus of management is on providing supportive care, which may involve implementing a ketogenic diet, administering CoQ supplements, and avoiding medications, such as valproate that could exacerbate mitochondrial dysfunction [18,19].

## 3. Mitochondrial Dysfunction and Aging

As we age, the physiological function of cells, tissues, and organs steadily deteriorates, leading to a reduced ability to adapt and repair. The process of aging is multifaceted and complex. Mitochondrial dysfunction, closely associated with the imbalance of the MQC system, is believed to be one of the contributing reasons to aging. Mitochondrial quality control (MQC) is an intrinsic cellular mechanism that encompasses an extensive network responsible for checking the integrity of mitochondria [20]. The system, essential for maintaining the balance and operation of mitochondria, comprises mitochondrial dynamics, autophagy, and biogenesis.

### 3.1. Mitochondrial Dynamics

“Mitochondrial dynamics” refers to the continuous process of division and fusion that occurs within the mitochondria. This activity is necessary to maintain the distribution, shape, and function of the mitochondria within the cell. Drp1, a GTPase kinetic protein-associated protein 1, facilitates the process of mitochondrial division. On the other hand, mitochondrial fusion is aided by the GTPase mitoproteins Mfn1 and Mfn2, as well as Opa1 [21,22,23]. Abnormal mitochondrial dynamics can also expedite cellular senescence. Studies have shown that in older mesenchymal stem cells, there is a decrease in the expression of DRP1 and an increase in the production of MFN2, which promotes the fusing of mitochondria. In contrast, conflicting studies demonstrated that the level of DRP1 was increased and the expression of OPA1 was reduced in the brains of aged mice [24,25]. Therefore, in order to maintain mitochondrial health and delay the aging process, it is crucial to maintain a state of equilibrium between mitochondrial fission and fusion.

### 3.2. Mitophagy

**Figure 2:**
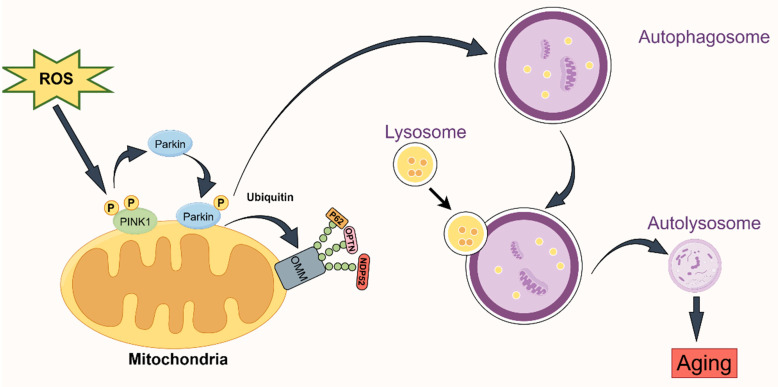
Mitophagy mediated by the PINK1–Parkin pathway during aging. PINK1 accumulates on the outer membrane of the mitochondria under depolarization or stress, and autophosphorylation activates it. In addition, the active PINK1 draws the cytoplasmic Parkin protein to the mitochondria and triggers Parkin’s E3 ubiquitin ligase activity by phosphorylating ubiquitin, which polyubiquitinates the protein found in the mitochondrial membrane. Specifically, signals for the identification of autophagy receptors are provided by ubiquitin chains connected by K63, and autophagy receptor proteins, like p62, OPTN, and NDP52, are attracted to mitochondria modified by ubiquitination to facilitate selective autophagy.

This form of autophagy is distinct. When mitochondria are injured, they are particularly enclosed in the autophagosome and combined with the lysosome. This completes the process of lysosome degradation, which helps maintain the balance and stability of mitochondria and cells and prevents damaged mitochondria from causing harm to cells. Mitophagy is crucial for regulating the quality of mitochondria. Both ubiquitin-dependent and non-ubiquitin-dependent processes contribute to mitophagy. The ubiquitin-dependent process, also known as Pink1–Parkin-mediated mitophagy, is facilitated by the two crucial proteins, PINK1 and Parkin [26,27]. Parkin functions as an E3 ubiquitin ligase, facilitating the transfer of ubiquitin to mitochondrial substrates. On the other hand, PINK1 is a serine/threonine kinase that is located on depolarized mitochondria. PINK1’s entry into the inner mitochondrial membrane is hindered by damage to the mitochondrial membrane potential. Consequently, PINK1 accumulates progressively on the cytoplasmic surface of the outer mitochondrial membrane. This process both attracts and activates Parkin, causing it to modify the spatial conformation of the Parkin protease into an activated E3 ubiquitin ligase. As a result, the protein on the mitochondria is ubiquitinated. Parkin and PINK1 collaborate to regulate mitophagy. Recent study suggests that mitophagy may activate the PINK/Parkin pathway, thereby accelerating the aging process [28,29,30] (Figure 2).

### 3.3. Mitochondrial Biogenesis

Mitochondrial biogenesis is the cellular process responsible for maintaining a consistent number of mitochondria by replacing damaged or aging mitochondria with new ones. The genes in both nDNA and mtDNA work together to govern the process of mitochondrial biogenesis. Co-regulatory transcription factors encompass PGC-1β, which stands for peroxisome proliferator activating receptor-gamma coactivator 1-α, as well as PGC-1β itself. They enhance the transcription of genes associated with respiration and the creation of new mitochondria, hence facilitating the fulfilment of metabolic requirements through the production of ATP [31,32,33].

## 4. ROS

ROS will be overexpressed in cells as a result of an imbalance in the MQC system. Both exogenous sources and the ETC generate ROS. Under normal physiological conditions, both enzyme-mediated and non-enzyme-mediated antioxidants can convert reactive oxygen species into less harmful byproducts. Excessive exposure to ROS leads to oxidative stress, resulting in protein oxidation and mutations in mitochondrial DNA [34,35,36]. Abnormal products in the mitochondria lead to an increase in the AMP/ATP ratio and a decrease in ATP production. Studies have shown that AMPK plays a vital role in controlling the aging process by promoting the creation of new mitochondria and ensuring their proper functioning [33,37,38]. Conversely, a continuous decrease in ATP leads to abnormal energy consumption, impeding the process of autophagy in the mitochondria, decreasing the production of new mitochondria, and impacting mitochondrial dynamics. These abnormal changes ultimately lead to cellular senescence and death.

## 5. Mitochondrial Dysfunction and Neurodegenerative Diseases

Neurodegenerative diseases are a collection of disorders that include the gradual deterioration and death of nerve cells, resulting in a reduction in cognitive function and other neurological problems. The disorders encompass Alzheimer’s disease (AD), Parkinson’s disease (PD), Huntington’s disease (HD), and others. Neurons are particularly vulnerable to damage and mortality caused by mitochondrial dysfunction because of their elevated energy demand. Reduced mitochondrial dynamics and function play a crucial role in the aging process and the onset of neurodegenerative diseases [39].

### 5.1. Alzheimer’s Disease (AD)

Alzheimer’s disease (AD) is rapidly becoming the leading cause of dementia, making it one of the most expensive, deadly, and debilitating illnesses of this century. Alzheimer’s disease is a progressive neurological disorder characterized by behavioral abnormalities, memory impairment, and cognitive deterioration [40]. Alzheimer’s disease is characterized by the presence of extracellular amyloid-beta (Aβ) plaques, intracellular neurofibrillary tangles composed of hyperphosphorylated tau protein, and neuronal death, which leads to brain atrophy and cognitive decline [41]. Prior research has shown that abnormal mitochondria in brain cells, characterized by irregular morphology, quantity, structure, and function, as well as altered mtDNA gene expression, are observed in individuals with Alzheimer’s disease. A study revealed that AD model mice exhibited minuscule and fragmented mitochondria in their brains, along with reduced respiration and mitochondrial membrane potential. Additionally, there was an elevated production of ROS [42,43,44]. Furthermore, the absence of mitochondrial ubiquitin ligase can worsen cognitive decline by causing mitochondrial harm and contributing to the disruption of mitochondrial dynamics and function observed in Alzheimer’s disease. Furthermore, research has demonstrated that the direct impact of Aβ on mitochondria might result in irregularities in mitochondrial coding genes. Therefore, Aβ could potentially interfere with mitochondrial function and contribute to the metabolic irregularities and neurological impairment observed in the brains of individuals with Alzheimer’s disease. Moreover, there is a continuous association between oxidative stress and Alzheimer’s disease. In cultured rat neurons, the presence of Aβ leads to the binding of 4-hydroxynonenal (HNE), an aldehyde produced during lipid peroxidation, to GLUT3. This binding inhibits the function of GLUT3 and reduces the levels of ATP, leading to mitochondrial malfunction. Consequently, this can trigger oxidative stress. Alternatively, it is plausible that mitochondrial dysfunction is an initial event that triggers the development of AD pathology, as mitochondrial biogenesis regulates the production of mitochondrial Aβ. Improving and sustaining the energy production of mitochondria, decreasing oxidative stress, and boosting mitochondrial activity can all contribute to combating AD [45,46,47,48,49].

### 5.2. Parkinson’s Disease (PD)

**Figure 3:**
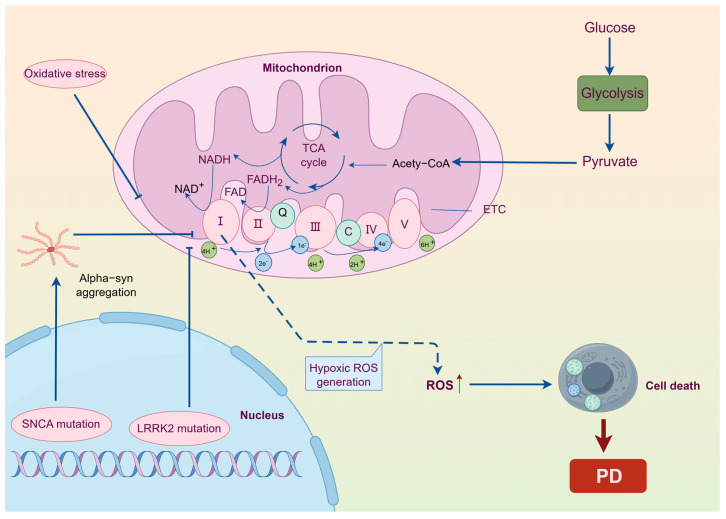
Complex regulatory networks in Parkinson’s disease. Parkinson’s disease has a complex etiology that includes both genetic and environmental influences. The three that will most likely impact mitochondrial function, cause abnormalities in mitochondrial electron transfer and oxidative phosphorylation, produce reactive oxygen species (ROS), and ultimately result in nerve cell death are oxidative stress, SNCA mutation, and LRRK2 mutation.

Parkinson’s disease (PD) is a common neurological disorder that affects individuals in the middle and later stages of life. It is characterized by a combination of motor and non-motor symptoms. The main motor symptoms include static tremor, myotonia, bradykinesia, and postural balance disruption, with slow movement being the most significant [50]. Non-motor symptoms encompass a range of conditions, such as depression, anxiety, dementia, impaired sense of smell, reduced salivation, difficulty swallowing, constipation, frequent urination, insomnia, excessive daytime sleepiness, and weariness. The focus of research on Parkinson’s disease has been mostly on its aetiology [51,52]. In 1989, Schapira and colleagues identified a deficiency in mitochondrial complex I in the brains of individuals with PD, suggesting that mitochondrial dysfunction contributes to the development of PD [53,54]. The improper use of α-synuclein (α-syn) is associated with the atypical accumulation and dysfunction of the mitochondria. α-syn oligomers can bind to mitochondrial ATP synthase, affecting the activity of the respiratory chain, promoting the peroxidation of mitochondrial lipids, causing the opening of the mitochondrial permeability conversion pore (MPTP), initiating the cascade of apoptosis, and finally leading to the death of nerve cells [55]. Moreover, deviations in the configuration and operation of the mitochondria are more conspicuous in individuals with Parkinson’s disease and deteriorate as they grow older. Parkinson’s disease can arise from various factors, such as exposure to environmental toxins, mutations and deletions in mtDNA, abnormal expression of genes associated with PD, and genetic mutations [56]. The unifying feature among all of these conditions is varying degrees of mitochondrial dysfunction. PD is characterized by the depletion of dopamine in the substantia nigra. Studies have shown that the substantia nigra is particularly susceptible to mitochondrial complex I dysfunction compared to other regions of the brain. Abnormal expression of mitochondrial complex I leads to a decrease in ATP synthesis, which in turn increases the production of ROS. This oxidative stress ultimately causes the death of dopamine neurons in the substantia nigra [57] (Figure 3).

### 5.3. Huntington’s Disease (HD)

Huntington’s disease (HD) is a hereditary neurological condition that occurs due to an aberrant increase in the number of CAG repeats in the huntingtin (HTT) gene [58]. The disease predominantly impacts the striatum and cerebral cortex, resulting in motor impairments, cognitive deterioration, and aberrations in mental behaviour [59]. Mitochondrial dysfunction is a significant factor in the development of HD and is regarded as one of the initial pathological alterations in HD. Studies have shown that HD cells experience significantly increased levels of oxidative stress, resulting in damage to the DNA of mitochondria and ultimately compromising the viability of nerve cells [60]. Furthermore, research has demonstrated that the balance of Ca^2+^ within the mitochondria is also disrupted in these cells [61,62]. The progress of HD is also associated with alterations in mitochondrial homeostasis, such as abnormal mitochondrial fusion and division [63]. Heat shock factor 1 (HSF1) perhaps plays a role in regulating disease processes and mitochondrial function in HD. HSF1 can influence mitochondrial function via regulating transcription factors, such as p53 and PGC-1α, which are associated with both mitochondrial activity and apoptosis [64]. Consequently, HSF1 may become a novel target for Huntington’s disease therapy.

## 6. Mitochondrial Dysfunction and Cardiovascular Diseases

Mitochondrial dysfunction has been significantly associated with the development of various cardiovascular illnesses, including diabetic cardiomyopathy, ischemia damage, and heart failure.

Mitochondrial dysfunction in heart failure is characterized by increased oxidative stress, dysregulated calcium homeostasis, and reduced energy output. Oxidative phosphorylation is the process by which the respiratory chain, a group of protein complexes located in the inner mitochondrial membrane, is responsible for generating the majority of ATP [65]. In cases of heart failure, the activity of these complexes is often compromised, resulting in reduced ATP synthesis and increased production of ROS, which can cause additional damage to the mitochondria of the cell. At the same time, the heart has the highest concentration of mitochondria of all human tissues, and a high concentration of mitochondria is essential to the heart’s ability to produce energy [66]. In a healthy adult heart, the main substrate for energy production is mitochondrial fatty acid oxidation (FAO) [67]. In heart failure, mitochondrial FAO is decreased and progressively switched to the glycolytic pathway. Finding possible molecular processes and treatment options for heart failure can be aided by researching mitochondrial anomalies in heart failure [68]. When a coronary artery has a sudden blockage, it can result in ischemic damage, which triggers a sequence of events involving tissue oxygen deprivation and cellular depletion of ATP. While reperfusion aids in the restoration of blood flow, it is important to note that ischemia–reperfusion injury (IRI) can actually exacerbate tissue damage. Multiple cellular components, such as mitochondria, participate in the molecular mechanisms that lead to IRI. Reperfusion can lead to an unregulated cascade mediated by ROS, which speeds up tissue necrosis upon the reintroduction of oxygen. Myocardial damage mostly occurs due to mitochondrial dysfunction caused by IRI, and additional damage and cell death are caused by the opening of the mitochondrial permeability transition pore (MPTP) [69,70,71].

Metabolic issues associated with increased levels of fatty acids in the bloodstream and heightened accumulation of fatty acids inside heart muscle cells are connected to diabetic cardiomyopathy. The persistence of the fatty acid oxidation (FAO) pathway in diabetic hearts is a significant factor contributing to reduced cardiac efficiency and contractile dysfunction, which are key features of diabetic cardiomyopathy. The excessive production of uncoupling proteins has a detrimental effect on the storage of calcium and the energy production of mitochondria, which worsens the condition of the mitochondria in diabetic cardiomyopathy [72]. Diabetes causes metabolic disruptions that result in progressive harm to the mitochondria, leading to an elevated production of ROS.

There is a significant amount of attention focused on treatments for cardiovascular illnesses that specifically address issues with mitochondrial dysfunction. These strategies involve creating antioxidants that particularly target the mitochondria and using antioxidants to decrease the production of reactive oxygen species and enhance their removal from the body. During preclinical studies, MitoTEMPO, mitoQ, and SS-31/MPT-131 have shown potential in reducing oxidative stress, improving mitochondrial function, and delaying the progression of cardiac disease [73,74,75]. Due to their involvement in energy generation, regulation of oxidative stress, and facilitation of cell death signaling, mitochondria play a vital part in the development and advancement of cardiovascular diseases. Directing interventions towards addressing mitochondrial dysfunction has the potential to serve as a therapeutic strategy for treating a range of disorders. Additional study is required to enhance the dosage, timing, and duration of treatment, as well as to understand the specific illness conditions in which these therapies may have the greatest efficacy, prior to their clinical application.

## 7. Mitochondrial Dysfunction and Metabolic Diseases

Disruptions in the body’s biochemical processes can lead to the accumulation or deficiency of certain metabolic chemicals, including carbohydrates, lipids, proteins (amino acids), purines, pyrimidines, and copper. This leads to metabolic disorders. The severity of symptoms varies, and the diagnosis is made by clinical indicators and biochemical tests, including blood and urine analysis. Currently, the available options for addressing the condition are limited to managing symptoms and identifying and removing the underlying cause. Unfortunately, there is no known remedy that can effectively eliminate the condition. The prognosis is influenced by various factors, such as the cause, severity of symptoms, and response to treatment. Obesity, diabetes, and fatty liver are prevalent metabolic disorders. Gaining a thorough comprehension of the process by which mitochondrial dysfunction operates in different diseases is beneficial for devising novel treatment approaches and therapies. The relationship between mitochondrial malfunction and the development and advancement of metabolic disorders is intricate.

## 8. Obesity

Non-alcoholic fatty liver disease (NAFLD) shares similar epidemiological and pathophysiological characteristics with prevalent metabolic conditions, such as obesity and type 2 diabetes mellitus (T2DM). The three main features of this condition, namely insulin resistance, altered metabolic flux, and ectopic fat deposition, are associated with abnormal energy metabolism. A recent study revealed a correlation between obesity and the activation of RalA, a small GTPase that plays a role in various cellular activities. Obesity leads to the activation of RalA, resulting in the dephosphorylation of the Drp1 protein. This, in turn, promotes mitochondrial division and dysfunction. The dephosphorylation of the inhibitory Serine637 site on the Drp1 protein and the subsequent activation of Drp1 occur due to the interaction between RalA and protein phosphatase 2. This connection leads to an excessive division and fragmentation of the mitochondria. This mitochondrial dysfunction may have a profound impact on the development of obesity and its consequences. Furthermore, alterations in mitochondrial activity in adipose tissue can result in the malfunction of adipose tissue, leading to compromised storage of triglycerides mediated by insulin and consequent leakage of lipids into other tissues. This can promote the occurrence and progression of steatosis, which can evolve from non-alcoholic fatty liver disease (NAFL) to non-alcoholic steatohepatitis (NASH) and liver fibrosis/cirrhosis [76].

### 8.1. Non-Alcoholic Fatty Liver Disease (NAFLD)

Non-alcoholic fatty liver disease (NAFLD) is a common chronic liver condition characterized by the accumulation of fat in the liver, unrelated to alcohol usage. The exact aetiology of NAFLD remains uncertain; however, mitochondrial dysfunction plays a crucial role in the illness’s pathophysiology. The fundamental role of liver mitochondria is to oxidize substrates, including fatty acids, pyruvate, and amino acids, in order to generate energy. NAFLD development has been associated with heightened production of ROS in the mitochondria and reduced oxidative phosphorylation. Moreover, changes in the number and activity of oxidative phosphorylation (OXPHOS) proteins are symptomatic of structural and molecular modifications in liver mitochondria in NAFLD, leading to decreased liver function [77].

### 8.2. Type 2 Diabetes Mellitus (T2DM)

Type 2 diabetes (T2DM) is a systemic disease characterized by high blood sugar, hyperlipidemia, and biochemical insulin resistance. The maintenance of normal blood glucose levels depends on the complex interplay between skeletal muscle and liver insulin sensitivity and the pancreatic beta cell’s secretion of glucose-stimulated insulin. The deficiency in the former is the cause of insulin resistance, while the latter’s deficiency leads to progression to hyperglycemia. Emerging evidence supports a potentially unifying hypothesis that both prominent features of T2DM are caused by mitochondrial dysfunction. Meanwhile, within the context of T2DM, various organs in the body exhibit structural, functional, and molecular changes in mitochondrial function, such as mitochondrial dysfunction in the digestive system, musculoskeletal system, and cardiovascular system [78,79,80]

## 9. Mitochondrial Dysfunction and Other Diseases

Mitochondria have a significant role in various diseases, including cancer, inflammatory conditions, and rare genetic disorders because of their involvement in immune response modulation, metabolic equilibrium, energy generation, and cell death control [81,82]. Mitochondria are recognised for their role in bioenergy production and supplying the necessary components for tumor growth, and for their involvement in the synthesis of molecules essential for cell growth and the regulation of cell death pathways. This connection is associated with the promotion of the Warburg effect, which is commonly observed in various types of cancers. Concurrently, cancer cells often exhibit mtDNA alterations. These mutations affect the efficiency of the electron transport chain, leading to increased production of ROS which in turn promote genetic instability and accelerate the growth of tumors. Due to their role in controlling the release of pro-apoptotic chemicals, such as cytochrome c, mitochondria also play a part in the resistance of cancer cells to apoptosis. Our understanding of the complex connections between mitochondria and cancer cell metabolism is crucial for the development of novel anticancer drugs. In the context of breast cancer, metformin hinders the proliferation of tumor cells by affecting mitochondrial metabolism via a decrease in insulin levels. In contrast, insulin promotes the proliferation of breast cancer cells while inhibiting the progression of the tumor by obstructing the mitochondrial complex I and PI3K pathways. Gboxin is a novel OXPHOS inhibitor, which is a type of drug that targets the activity of the mitochondrial F0F1 ATP synthase with high specificity. It is a small molecule compound [83]. Gboxin inhibits the proliferation of normal cells and suppresses the growth of glioblastoma cancer stem cells [84]. Gboxin in liver cancer disrupts the interaction between TOMM34 and ATP5B, resulting in a decrease in ATP production and a reduction in the migration of cancer cells. This approach synergistically complements the action of metformin to effectively treat liver cancer [85].

Mitochondria have a multifaceted role in inflammation, acting as both an effector and a regulator of the inflammatory response. The mtDNA is thought to substantially activate the cyclic GMP-AMP synthase (cGAS) pathway and the stimulator of interferon genes 1 (STING1) signaling. This signaling pathway leads to the production of cytokines, such as IL-6, TNF-β1, and interferon-β1 (IFN-β1). Moreover, mtDNA possesses the capacity to trigger the inflammasome complex, an essential component for the production of IL-1β and IL-18, two crucial cytokines involved in inflammation [86]. The immune system’s response to stress or infection can be initiated by the release of mtDNA, which then interacts with immunological receptors, such as cGAS and inflammasome, leading to a sequence of inflammatory processes. Meanwhile, the obstruction of voltage-dependent anion channels (VDAC) or the inhibition of mitochondrial respiratory chain complex I or III hinders the activation of the NLRP3 inflammasome [87]. In addition, inflammation can be triggered by mitochondrial dysfunction and the subsequent release of mitochondrial components, such as formylated peptides and cardiolipins, through Toll-like receptor 9 (TLR9) and other pattern recognition receptors (PRRs) [88]. To generate an effective immune response, these interactions can trigger the activation of immune cells and the production of inflammatory mediators.

## 10. Therapy

The diagnosis and management of mitochondrial malfunction, a complex matter that can lead to various disorders, are constantly evolving (Table 2). The current methods for identifying mitochondrial disorders encompass genetic testing, biochemical testing, clinical assessment, and, in some cases, tissue biopsy. Research conducted on blood and urine has found that elevated lactate levels in the cerebrospinal fluid can serve as a reliable diagnostic marker for mitochondrial sickness in individuals who also have neurological symptoms [89]. Signs of mitochondrial malfunction, such as elevated levels of lactic acid and pyruvate, are detected using biochemical screening assays in both blood and urine. Additionally, urine organic acid analysis and amino acid analysis are performed to further assess mitochondrial activity [90]. Genetic testing can identify the specific molecular cause of a certain type of mitochondrial myopathy that is caused by mutations in either mtDNA or nDNA. This testing can confirm the diagnosis and guide the selection of appropriate therapeutic interventions, such as the occurrence of MELAS caused by MTTL1 mutations [91,92,93]. Currently, the primary approaches to treating mitochondrial diseases focus on providing support and prevention, with a particular emphasis on managing symptoms and addressing the consequences of the illness. Specific therapies, such as coenzyme Q10, thiamine, riboflavin, biotin, or niacin supplements, can be used to treat certain cases of genetic cofactor shortages [94,95]. In addition, several drugs, such as idebenone, have received approval for the treatment of LHON [96,97]. However, there is currently disagreement regarding their efficacy in this context. Moreover, significant progress has been made in developing innovative treatments for diseases associated with mitochondria. These include vitamin and mineral supplements, physical exercise, medication, tissue replacement, gene therapy, and other similar treatments. Ultimately, conducting research on mitochondrial dysfunction in disease treatment is crucial for developing targeted therapies that improve patient outcomes [98,99,100,101,102,103]. Current research and clinical trials offer hope for the development of viable therapeutics for a range of illnesses caused by mitochondrial malfunction.

## 11. Conclusions

Mitochondria are essential organelles within cells, playing critical roles not only in energy metabolism but also in various cellular activities, such as cell differentiation, signal transduction, and apoptosis. Mitochondrial dysfunction is implicated in a range of diseases, including but not limited to diabetes and its complications, neurodegenerative disorders, myocardial ischemia–reperfusion injury, and heart failure. Therefore, investigating the structure and function of mitochondria as well as the mechanisms underlying mitochondrial dysfunction in disease contexts holds significant scientific and clinical importance.

Basic scientific research: Diseases manifest systemically and exhibit complexity; thus, it is imperative to understand mitochondrial structure at the molecular level along with known pathways while characterizing novel pathways that influence mitochondrial behavior and functionality. For instance, mapping genetic interactions among genes encoding mitochondrial proteins can elucidate interrelations between different aspects of mitochondrial function. The first focused map of mitochondria has been constructed in yeast models, revealing dense and significant connections among localization pathways distributed across various mitochondrial compartments [126].

Disease diagnosis: A comprehensive understanding of the mechanisms governing mitochondrial dysfunction can facilitate the development of innovative diagnostic tools. By monitoring specific indicators related to mitochondrial function, earlier diagnosis of diseases associated with mitochondrial impairment becomes feasible. Employing next-generation sequencing technologies for analyzing the mitochondrial proteome aids in identifying novel proteins and pathways linked to mitochondria while enabling streamlined diagnostics alongside genetic counseling opportunities for patients with mitochondrial diseases [127].

Drug development: Advancements in our comprehension of how mitochondria contribute to disease processes may promote targeted therapeutic strategies. For example, metformin—a widely used antidiabetic agent—has recently been repurposed as an anticancer drug; its combination with standard epidermal growth factor receptor tyrosine kinase inhibitors (EGFR-TKIs) significantly improves progression-free survival rates and overall survival outcomes for patients with advanced lung adenocarcinoma [125].

Personalized medicine: Given that manifestations of mitochondrial dysfunction may vary among individuals, research into mitochondria provides a theoretical foundation for personalized medicine by allowing tailored treatment plans based on individual states of mitochondrial functionality [127].

Furthermore, elucidating interaction mechanisms between mitochondria and other organelles (such as the endoplasmic reticulum, nucleus, and lysosomes) remains an area requiring further investigation. These inter-organelle interactions play crucial roles in maintaining cellular homeostasis while regulating metabolism under stress conditions through signaling pathways. Future research into these intricate relationships will yield deeper insights into cellular functions as well as disease mechanisms, potentially unveiling new therapeutic targets.

## Figures and Tables

**Table 1:** Genetic classification of mitochondrial diseases.

Defect Location	Mode of Inheritance	Genetic Diseases and Their Characteristics	Biochemical Analysis
nDNA defect			
Tissue-specific genes	Mendelian	Tissue-specific syndrome	Tissue-specific single enzyme lesion
Non tissue-specific genes	Mendelian	Multisystem disease	Widespread enzymatic lesions
mtDNA defect			
Point mutation	Maternal inheritance	Multiple systems, impurities	Specific single enzyme lesion Widespread enzymatic lesions
Deletion	Distribute	PEO, KSS, Pearson	Widespread enzymatic lesions
NDNA and mtDNA combined defects			
Multiple mtDNA deletions	AD/AR	PEO	Widespread enzymatic lesions
MtDNA deletion	AR	Myopathy, liver disease	Tissue-specific multi enzyme lesions

Note: PEO: progressive extraocular muscle paralysis; KSS: ophthalmic myopathy; Pearson: bone marrow/pancreatic syndrome; AD: autosomal dominant; AR: autosomal recessive.

**Table 2:** Typical mitochondrial diseases.

Diseases	Mitochondrial Targeted Therapy	References
mtDNA mutation		
Leber’s inherited optic neuropathy	Idebenone, EPI-743	[13,104,105,106]
Myoclonic epilepsy with red ragged fibers	Levetiracil is used in combination with benzodiazepines, such as clonazepam or clobazam	[107,108]
nDNA mutation		
Leigh syndrome	There is no radical treatment for SURF1, and the ketogenic diet is the most prescribed treatment	[17,109,110]
Neurodegenerative Diseases		
Alzheimer’s disease	Latrepyridine, ketogenic diet	[111,112]
Parkinson’s disease	Some natural drugs, such as phenol, alkaloids, flavonoids, terpenoids, and saponins, are expected to be mitochondria-targeted drugs for Parkinson’s disease. LRRK2 inhibitors in clinical trials hold great promise in correcting mitochondrial dysfunction and mitochondrial autophagy defects outside of patients with LRRK2 mutations	[113,114]
Huntington’s disease	MTAXs (SS31, CDDO-ethyl amide, XJB-5–13, MitoQ, bezafibrate, rosiglitazone, meldonium, and coenzyme Q10) may be a beneficial target for mitochondrial homeostasis and may be used as a therapeutic strategy for HD	[115]
Cardiovascular diseases		
Heart failure	Omecamtiv mecarbil, ivabradine	[116]
Ischemia–reperfusion injury	Idebenone, natural plant compounds: luteolin, resveratrol, fraxetin, puerarin, baicalein, araloside total saponins, and araloside A	[117,118]
Diabetic cardiomyopathy	Sotagliflozin, empagliflozin	[119]
Metabolic diseases		
Obesity	Physical activity, dinitrophenol	[120,121]
Non-alcoholic fatty liver Disease	Vitamin E, pioglitazone	[122]
T2DM	Metformin, physical activity	[123]
Cancer	Ketogenic diet, metformin, CPI-613	[123,124,125]
